# The impact of radiotherapy on disease control in vulvar extramammary Paget’s disease: a retrospective study from a single institution

**DOI:** 10.3389/fonc.2025.1520528

**Published:** 2025-02-12

**Authors:** Aeran Seol, Se Ik Kim, Yong Sang Song

**Affiliations:** ^1^ Department of Obstetrics and Gynecology, Seoul National University Hospital, Seoul, Republic of Korea; ^2^ Department of Obstetrics and Gynecology, Myongji Hospital, Goyang-si, Gyeonggi-do, Republic of Korea

**Keywords:** extramammary Paget’s disease, radiotherapy, recurrence, postoperative complication, neoadjuvant radiation therapy, adjuvant radiation therapy

## Abstract

**Objective:**

Vulvar extramammary Paget’s disease (EMPD) is a rare intraepithelial carcinoma that affects apocrine gland-bearing skin, predominantly in postmenopausal women. Due to its rarity, optimal treatment strategies, including the role of radiotherapy (RT), remain poorly established. This study aimed to evaluate the role of radiation therapy in vulvar EMPD, focusing on preserving functional and aesthetic vulvar tissue without compromising survival rates.

**Materials and methods:**

We conducted a retrospective cohort study of 32 patients diagnosed with vulvar EMPD at Seoul National University Hospital between 2000 and 2024. Clinicopathologic data, including demographics characteristics, clinical presentations, histopathological findings, treatment modalities, and outcomes, were collected. Patients were divided into two groups: those who received neoadjuvant or adjuvant RT (*n* = 9) and those who did not (*n* = 21). Univariate and multivariate analyses were performed to assess factors related to recurrence and progression-free survival (PFS).

**Result:**

The median age at diagnosis was 63.8 years (range: 38.0–87.8), with 84.4% of patients being postmenopausal. Among the 32 patients, 30 (93.8%) underwent surgery, and nine (28.1%) received adjuvant RT. Recurrence rates were similar between the RT (66.7%) and non-RT (66.7%) groups. The median PFS was longer in the RT group (28.1 months) compared to the non-RT group (23.4 months), although this difference was not statistically significant (*p* = 0.395). Univariate and multivariate analyses identified age ≥ 60 years as a borderline predictor of poorer PFS (*p* = 0.053), while no significant associations were found between RT and postoperative complications or recurrence risk.

**Conclusion:**

In conclusion, although RT did not show a statistically significant survival benefit, both our data and previous studies strongly suggest that RT holds potential for disease control. It may be the primary treatment before and after surgery in patients with extensive vulvar EMPD.

## 1 Introduction

Extramammary Paget’s disease (EMPD) is a rare intraepithelial carcinoma that affects apocrine gland-bearing skin (1). Primary EMPD develops from the epidermis, while secondary EMPD arises from other underlying malignancies. The most common form of EMPD is vulvar Paget’s disease (VPD), which predominantly affects postmenopausal women over the age of 60 years (2, 3). The most common location for vulvar EMPD is the labia majora, where the lesions consist of erythematous plaques or eczema-like skin changes (4). Due to its low incidence, the clinical course, optimal management, and prognosis of vulvar EMPD are not as well-defined as those of other vulvar malignancies, posing a challenge to clinicians.

The primary treatment modality for vulvar EMPD is surgical excision, with the goal of achieving complete removal of the disease (5). Due to its indolent nature, the prognosis is generally favorable, with a 5-year survival rate of 75%–90% (6). However, despite this positive prognosis, the local recurrence rate after surgery is 15%–61%, and the risk factors for recurrence remain controversial (7). The pathological margins of vulvar EMPD often extend beyond visible clinical boundaries, which complicates the challenge of achieving complete surgical excision (8). This issue is related to the extensive surgical procedures required, which can cause significant morbidity, including functional and cosmetic defects that may negatively impact patient quality of life and outcomes (9). These limitations have driven interest in alternative and adjunctive therapies, including radiotherapy (RT), which has shown promise as a noninvasive treatment option, particularly for patients who are not ideal surgical candidates, such as the elderly (6).

Radiotherapy’s role in managing EMPD has been increasingly recognized, with evidence suggesting it can offer excellent local control and palliation with minimal toxicity (10–13). However, the data on the efficacy and safety of RT specifically for vulvar EMPD remain limited, with most available literature consisting of case reports and small retrospective series. Additionally, vulvar EMPD predominantly affects Caucasian women, resulting in a significant lack of research focused on Asian women (14). This gap in comprehensive studies underscores the need for further research on the long-term outcomes of RT in the treatment of vulvar EMPD.

This paper aims to conduct a retrospective cohort study to examine the treatment and outcomes of patients with vulvar EMPD, specifically focusing on those who have undergone radiotherapy. By analyzing these patients’ outcomes, we aim to evaluate the effectiveness of radiotherapy as a potential treatment option.

## 2 Materials and methods

This study was conducted as a single-center, retrospective analysis of patients diagnosed with vulvar EMPD at Seoul National University Hospital from 2000 to 2024. The institutional Review Board of Seoul National University Hospital approved the study (No. 2407-102-1553). Eligible patients were identified through the hospital’s electronic medical records database. Inclusion criteria consisted of individuals aged 18 years or older who had been diagnosed with vulvar EMPD, confirmed by histopathological examination. Patients with incomplete medical records were excluded to ensure data integrity.

Data collection involved a comprehensive review of the medical records of identified patients. Information was collected, including demographic characteristics such as age of diagnosis, parity, and menopausal status, as well as clinical presentation, including symptoms, disease duration, and any complications after treatment. Additionally, histopathological findings, treatment modalities employed, and follow-up outcomes were meticulously documented.

To evaluate cases involving the use of neoadjuvant radiotherapy in the treatment of vulvar EMPD, a comprehensive literature search was performed across PubMed, EMBASE, Web of Science, Scopus, and Cochrane Library up to September 2024, using key terms such as “vulvar Paget” and “vulvar Paget’s”. The inclusion criteria focused on full-text, peer-reviewed articles published since 1990, primarily reporting on vulvar EMPD with treatment details and outcomes. Exclusion criteria included cases that focused solely on pathological, immunohistochemical, or molecular aspects.

Descriptive statistics were used to summarize the demographic and clinical characteristics of the study population. Continuous variables were reported as mean ± standard deviation or median with interquartile range, while categorical variables were presented as frequencies and percentages. For statistical analyses, Chi-square and Fisher’s exact test were used for categorical variables, and *t*-tests were applied to continuous variables. A significance threshold was a *p*-value of < 0.05 to determine statistical relevance. All analyses were conducted using SPSS 25.0 (IBM Corp., Armonk, NY, USA).

## 3 Results

A total of 32 patients diagnosed and treated for vulvar EMPD were included in this retrospective study. The median age at diagnosis was 63.8 years (range: 38.0–87.8). The most common presenting symptom was pruritus, affecting 46.9% of patients, followed by skin changes (37.5%), pain (6.3%), and a mass (6.3%). The majority of patients (84.4%) were postmenopausal, and 15.6% had a history of other malignancies. Among the patients, 30 (93.8%) underwent surgery, and nine (28.1%) received adjuvant or neoadjuvant radiotherapy. Concomitant chemoradiation therapy (CCRT) was administered in two patients (6.3%), and three patients (9.4%) received chemotherapy. Characteristics of the patients are shown in **Table 1**.

**Table 1:** Clinicopathologic characteristics of the study population.

Characteristics	All (*n* = 32)
Age at initial diagnosis (years)	63.8 (38.0–87.8)
	2 (0–6)
Symptoms	
Ulcer	1 (3.1)
Pruritis	15 (46.9)
Pain	2 (6.3)
Skin change	12 (37.5)
Mass	2 (6.3)
Menopause	
Yes	27 (84.4)
No	5 (15.6)
Other malignancy	
Yes	5 (15.6)
No	27 (84.4)
Treatment	
Surgery	30 (93.8)
Radiotherapy (RT)	9 (28.1)
Concomitant chemoradiation (CCRT)	2 (6.3)
Chemotherapy	3 (9.4)
Other	3 (9.4)
Recurrence	21 (65.6)
Death	2 (6.3)

We divided patients who underwent surgery into two groups to evaluate the impact of preoperative or postoperative radiotherapy on surgical complications and recurrence: one group of nine patients who received radiotherapy (RT group) and another group (non-RT group) of 21 patients who did not. When comparing the RT and non-RT groups, no significant differences were observed in terms of age at diagnosis, type of surgery, lesion size, or bilateral lesion involvement. Positive surgical margins were reported in 70.0% of the total cohort, with similar rates between the RT (66.6%) and non-RT (71.4%) groups (*p* > 0.999). The clinicopathologic characteristics of the two groups are summarized in **Table 2**.

**Table 2:** Clinicopathologic characteristics of surgical patients.

Characteristics	All (*n* = 30)	RT group (*n* = 9)	Non-RT group (*n* = 21)	*p*-value
Age at diagnosis (years)	62.4 (38.0–79.8)	59.8 (44.7–78.3)	63.5 (38.0–79.8)	0.893
Other malignancy	4 (13.3)	3 (33.3)	1 (4.8)	> 0.999
Type of surgery					
Simple vulvectomy	13 (43.3)	3 (33.3)	10 (47.6)	0.466
Wide local excision	9 (30.0)	2 (22.2)	7 (33.3)	
Radical vulvectomy	8 (26.7)	4 (44.4)	4 (19.0)	
Reconstruction surgery	11 (36.7)	4 (44.4)	7 (33.3)	0.687
Bilateral lesion	11 (36.7)	2 (22.2)	9 (42.9)	0.419
The largest diameter of the lesion	5.7 (2.2–12.1)	6.8 (2.2–11.0)	5.7 (2.4–12.1)	0.614
Positive surgical margin	21 (70.0)	6 (66.6)	15 (71.4)	> 0.999
Invasiveness	10 (33.3)	5 (55.6)	5 (23.8)	0.083
Adnexal involvement	20 (66.7)	6 (66.6)	14 (66.7)	> 0.999
Lymph node (LN) metastasis	4 (13.3)	2 (22.2)	2 (9.5)	0.563
Postoperative complications	6 (20.0)	2 (22.2)	4 (19.0)	> 0.999
Recurrence	7 (23.3)	6 (66.7)	14 (66.7)	> 0.999
Death	23 (76.7)	1 (11.1)	1 (4.8)	0.517

To identify factors associated with postoperative complications in patients who underwent surgery for vulvar extramammary Paget’s disease, a univariate analysis was performed. Although lesion size at ≥ 6 cm showed a trend toward a higher risk of postoperative complications with an odds ratio (OR) of 7.778 (95% CI: 0.776–77.931), this was not statistically significant (*p* = 0.081). Reconstruction surgery also showed a potential trend, with an OR of 4.857 (95% CI: 0.718–32.867), but this did not reach statistical significance (*p* = 0.105). Overall, no variable demonstrated a significant predictive value for the development of postoperative complications (**Table 3**).

**Table 3:** Univariate analyses for postoperative complication.

Variables	Odds ratio (95% CI)	*p*-value
Age ≥ 60	1.692 (0.259–11.065)	0.583
Lesion size ≥ 6.0 cm	7.778 (0.776–77.931)	0.081
Bilateral lesion	2.000 (0.327–12.238)	0.453
Invasiveness	0.417 (0.040–4.334)	0.464
Positive margin	0.706 (0.102–4.891)	0.724
History of radiation	1.214 (0.179–8.217)	0.842
Radical vulvectomy	1.5 (0.217–10.361)	0.681
Reconstruction surgery	4.857 (0.718–32.867)	0.105

**Figure 1:**
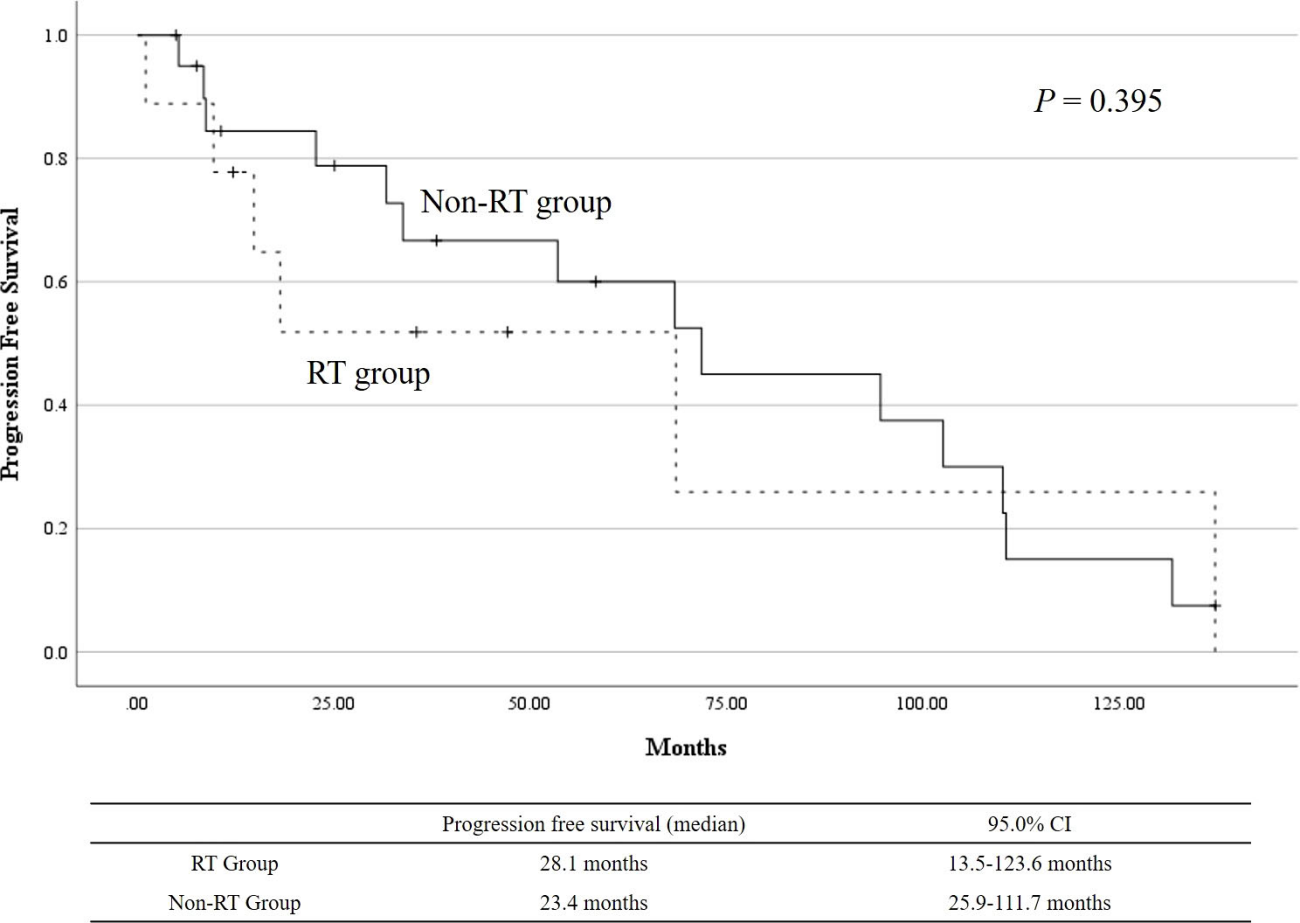
Kaplan–Meier curves for progression-free survival (PFS) in the RT and non-RT groups.

When we evaluated recurrence in the two groups, recurrence rates were comparable between the groups (RT: 66.7%, non-RT: 66.7%, *p* > 0.999) (**Table 4**). The median progression-free survival (PFS) in the RT group was 28.1 months (range: 13.5–123.6), while the median PFS in the non-RT group was 23.4 months (range: 25.9–111.7) (**Figure 1**). Despite a numerically longer PFS in the RT group, the difference between the two groups was not statistically significant (*p* = 0.395). This suggests that, while there may be a trend toward improved disease control in patients receiving RT, the survival benefit was not definitively established within this study cohort. Further analysis may be needed to clarify the role of RT in reducing recurrence risk.

**Table 4:** Univariate and multivariate analyses for progression-free survival.

Characteristics	Progression-free survival					
	Univariate analysis			Multivariate analysis		
	HR	95% CI	*p*-value	aHR	95% CI	*p*-value
Age at diagnosis ≥ 60	2.453	0.923–6.515	0.072	3.604	0.982–13.221	0.053
History of cancer	0.123	0.011–1.386	0.090	2.178	0.107–44.431	0.613
Lesion size ≥ 6.0 cm	0.250	0.480–1.292	0.098	0.272	0.053–1.389	0.118
Bilateral lesion	9.000	0.954–84.899	0.055	0.565	0.102–3.139	0.514
Invasiveness	1.361	0.263–7.035	0.713	0.913	0.205–4.069	0.905
Positive margin	1.200	0.220–6.534	0.833	2.544	0.364–17.757	0.346
Lymph node involvement	1.588	0.144–17.561	0.706	7.227	0.746–70.000	0.088
Reconstruction surgery	0.429	0.090–2.051	0.289	2.013	0.578–7.010	0.272
Preoperative RT	1.268	0.478–3.365	0.633	1.560	0.263–9.250	0.624

During the univariate and multivariate analyses for PFS, age at diagnosis ≥ 60 years (HR: 2.453, 95% CI: 0.923–6.515, *p* = 0.072) and bilateral lesions (HR: 9.000, 95% CI: 0.954–84.899, *p* = 0.055) showed trends toward being associated with shorter PFS in univariate analysis, although neither reached statistical significance. In the multivariate analysis, age ≥ 60 years remained a borderline predictor of poorer PFS (adjusted HR: 3.604, 95% CI: 0.982–13.221, *p* = 0.053), but no other factors, including lesion size, lymph node involvement, or history of cancer, were significant predictors of progression.

To evaluate the effectiveness of neoadjuvant radiotherapy, two specific cases are summarized in **Table 5**. Both patients received radiotherapy prior to surgery, resulting in negative surgical margins and only small wound dehiscence in case 2, indicating the potential benefit of neoadjuvant radiotherapy in achieving complete resection. PFS varied between the two cases.

**Table 5:** Summary of neoadjuvant radiotherapy in two cases of vulvar EMPD.

Case	Age at diagnosis	Duration of radiotherapy	Operation	Surgical margin status	Lesion size (cm)	Postoperative complication	Adjuvant treatment	Progression-free survivals
1	51	6 weeks	Radical vulvectomy	Negative	11.0 × 10.4	None	6 cycles of 5-FU-cisplatin	137.23
2	77	6 weeks	Local excision	Negative	10 × 5 (left) 1 × 1 (right)	2 cm wound dehiscence	None	9.69

Previously published cases of neoadjuvant radiotherapy in the treatment of vulvar EMPD are summarized in **Table 6**. Besa et al. reported that a 38-year-old patient received 29 days of radiotherapy followed by abdominoperineal resection; the patient experienced perineal wound necrosis but showed no evidence of local disease for 15 months (15). Cai et al. presented a 59-year-old patient who underwent pelvic external beam RT followed by two cycles of chemotherapy due to extensive retroperitoneal lymph node metastasis. The patient showed no recurrence following treatment, although no specific recurrence-free duration was reported (16). Yanagi et al. described an 86-year-old patient treated with 8 weeks of radiotherapy followed by surgical excision of the remaining lesion (17). The patient remained free of recurrence for 5 months.

**Table 6:** Summary of neoadjuvant radiotherapy cases in previous studies.

Case	Age at diagnosis	Duration of radiotherapy	Operation	Postoperative complication	Adjuvant treatment	Progression-free duration
Besa et al.	38	Radiotherapy for 29 days	Abdominoperineal resection: rectal adeno, perianal Paget’s disease	Perineal wound necrosis	Locally NED 15 months; died of disseminated rectal carcinoma	15 months
Cai et al.	59	Pelvic external-beam radiotherapy followed by 2 cycles of chemotherapy	Extensive retroperitoneal lymph node metastasis	NR	NR	No recurrence
Yanagi et al.	86	Radiotherapy during 8 weeks	Surgical excision of the remained lesion	NR	NR	No recurrence after 5 months

*NR*, not reported.

## 4 Discussion

This retrospective study examined the clinicopathologic characteristics and outcomes of 32 patients diagnosed with vulvar EMPD and evaluated the potential role of RT in improving disease control of 30 surgically treated patients.

In the analysis of factors related to postoperative complications, no association with RT was found. Of the nine patients in the RT group, two developed mild wound dehiscence, which was managed with antibiotics alone. No toxicities greater than grade 3 were observed in the RT group. Although lesion size ≥ 6 cm was not significantly associated with postoperative complications, it was evaluated as a trend toward a higher risk of complications. Several studies have evaluated clinical factors influencing postoperative complications in patients undergoing surgical treatment for vulvar EMPD. Cho et al. emphasized that increased tumor size and invasiveness are associated with wound healing issues (18). In some cases, vulvar EMPD involves extensive lesions, which may delay the initiation of surgical treatment. Li et al. and Tran and Harvey reported a case of extensive EMPD, highlighting how neoadjuvant chemoradiation effectively reduced tumor burden, enabling successful surgery (19, 20). Li et al. describe a patient who underwent neoadjuvant chemoradiotherapy followed by surgical excision, with no postoperative complication reported. Remarkably, they noted that the patient was able to achieve early discharge after surgery, suggesting a favorable postoperative course without complications. In contrast, neoadjuvant chemoradiation was not performed in our study.

For the evaluation of recurrence in the RT and non-RT groups, statistical significance was not achieved in the PFS analysis. The PFS in the RT group (median PFS of 28.1 months) was longer than that in the non-RT group (median PFS of 23.4 months), but the difference was not statistically significant (*p* = 0.395). Despite this, the numerically longer PFS in the RT group suggests a potential benefit of RT in reducing the recurrence risk in select patients. The role of radiotherapy in the management of vulvar EMPD remains poorly defined due to a lack of studies. Early studies reported high recurrence rates of up to 80% following RT, while more recent studies reported relatively lower rates, ranging from 0% to 35% (21–23). For adjuvant RT, it has been recommended for high-risk factors such as lymph node metastasis, positive surgical margins, and multifocal disease (13, 24, 25). These studies report recurrence rates ranging from 28.6% to 50%. In our study, a total of seven patients underwent adjuvant RT, and four patients experienced disease recurrence. Six patients were positive for surgical margins, and two patients had invasive disease. Several studies, including systematic reviews on RT, have been conducted, with the disease included in these studies varying in their spectrum. Additionally, there was diversity in RT techniques, such as dosing and fractionation, and remission rates varied widely, ranging from 50% to 100% (26). Compared to earlier studies, more recent research has gradually narrowed the range of dose and fractions, and results have been reported. However, a standard protocol has not yet to be determined. Due to the lack of standardized randomized controlled trials, additional research is needed to better understand the effectiveness of RT.

The effectiveness of neoadjuvant radiotherapy in treating vulvar EMPD is highlighted by two cases in our study, where both patients achieved negative surgical margins with only minimal postoperative complications, suggesting its potential role in facilitating complete resection. However, the variation in PFS underscores the need for further investigation into long-term outcomes. Previous studies have reported various results, despite the small number of cases. Besa et al. reported a patient who remained disease-free for 15 months, while Cai et al. and Yanagi et al. reported outcomes with no recurrence (15–17). Besa et al. also noted that the patient experienced postoperative complications. Although the follow-up periods in the reported cases are limited, the outcomes suggest that neoadjuvant radiotherapy can provide effective local control in cases of advanced vulvar EMPD, particularly when there is no spread to intra-abdominal organs. This indicates that neoadjuvant radiotherapy may play a crucial role in enhancing surgical resectability and improving overall disease management.

Our study focuses on the role of RT in treating vulvar EMPD, a topic that has been sparsely investigated in previous literature. Given the rarity of EMPD, few studies have specifically examined the potential benefits of RT, making this research a valuable contribution to the limited body of evidence regarding treatment options. By retrospectively analyzing real-world outcomes in patients treated at a single institution, this study provides important insights into the potential of RT.

However, the study has several limitations. First, due to the rarity of the disease, the cohort includes patients treated over a long period, which may introduce variability in treatment approaches and follow-up protocols. Additionally, differences in radiation protocols and the heterogeneity of the patient population—such as variations in disease severity, lesion size, and treatment modalities—pose challenges for the analysis and may limit the generalizability of the findings. The small sample size of the overall population and the RT group further complicate the ability to draw statistically significant conclusions, particularly regarding the effect of RT on PFS. Future studies with larger, more homogenous patient cohorts are needed to validate these findings and provide more definitive evidence on the role of RT in EMPD.

In conclusion, although this study did not demonstrate a statistically significant survival benefit for RT, the observed trends in our data suggest that RT may hold promise as a potential treatment option for improving disease control in vulvar EMPD without severe toxicities. Both our data and previous studies strongly indicate that RT could be considered the primary neoadjuvant therapy in patients with extensive vulvar EMPD.

## Data Availability

The raw data supporting the conclusions of this article will be made available by the authors, without undue reservation.
